# Supplementary material for: Meta-Analysis and Gene Set Enrichment Relative to ER Status Reveal Elevated Activity of MYC and E2F in the “Basal” Breast Cancer Subgroup
Source: PLoS One. 2009 Mar 9;4(3):e4710. doi: 10.1371/journal.pone.0004710 (PMC2650420; doi:10.1371/journal.pone.0004710)
Supplement: Dataset S1 — GSEA results from first screen. The file contains a zipped archive of the HTML results of the second GSEA screen. Reports for enriched or depleted datasets may be accessed from the “index.html” link within the my_analysis.GseaPreranked.1219975950625.rpt.zip ZIP archive. Enrichment plots are available for each of the 50 most enriched and depleted gene sets. (13.32 MB ZIP) [file pone.0004710.s001.zip › my_analysis.GseaPreranked.1219975950625.rpt.zip!//VERNELL_PRB_CLSTR2.html]

Details for gene set VERNELL\_PRB\_CLSTR2[GSEA]

|  || Dataset | zSco.ERneg.to.ERpos\_collapsed |
| Phenotype | NoPhenotypeAvailable |
| Upregulated in class | na\_neg |
| GeneSet | VERNELL\_PRB\_CLSTR2 |
| Enrichment Score (ES) | -0.53449404 |
| Normalized Enrichment Score (NES) | -1.7240791 |
| Nominal p-value | 0.0054347827 |
| FDR q-value | 0.05464561 |
| FWER p-Value | 0.95 |
Table: GSEA Results Summary

  

Fig 1: Enrichment plot: VERNELL\_PRB\_CLSTR2      
 Profile of the Running ES Score & Positions of GeneSet Members on the Rank Ordered List

  

| PROBE | GENE SYMBOL | GENE\_TITLE | RANK IN GENE LIST | RANK METRIC SCORE | RUNNING ES | CORE ENRICHMENT || 1 | ITGB8 |  |  | 354 | 4.777 | 0.0709 | No |
| 2 | CPA4 |  |  | 1033 | 3.433 | 0.0900 | No |
| 3 | RSNL2 |  |  | 1993 | 2.536 | 0.0697 | No |
| 4 | NMD3 |  |  | 5966 | 0.827 | -0.2121 | No |
| 5 | TMEPAI |  |  | 6257 | 0.729 | -0.2190 | No |
| 6 | TOB2 |  |  | 6969 | 0.499 | -0.2623 | No |
| 7 | PPFIBP1 |  |  | 7278 | 0.392 | -0.2774 | No |
| 8 | ZNF614 |  |  | 7402 | 0.345 | -0.2796 | No |
| 9 | SERPINE1 |  |  | 8947 | -0.283 | -0.3899 | No |
| 10 | CTGF |  |  | 9358 | -0.487 | -0.4108 | No |
| 11 | FAM86C /// FAM86B1 / |  |  | 9814 | -0.734 | -0.4300 | No |
| 12 | EHD3 |  |  | 10213 | -0.965 | -0.4403 | No |
| 13 | DYNLRB1 |  |  | 11411 | -1.943 | -0.4906 | Yes |
| 14 | CITED2 |  |  | 11937 | -2.484 | -0.4794 | Yes |
| 15 | SH3BP4 |  |  | 12671 | -3.558 | -0.4619 | Yes |
| 16 | GPRC5A |  |  | 13037 | -4.586 | -0.3957 | Yes |
| 17 | HIST2H2BE |  |  | 13073 | -4.726 | -0.3019 | Yes |
| 18 | DCTN4 |  |  | 13113 | -4.875 | -0.2053 | Yes |
| 19 | CCND1 |  |  | 13160 | -5.098 | -0.1047 | Yes |
| 20 | DIP13B |  |  | 13233 | -5.716 | 0.0065 | Yes |
Table: GSEA details [plain text format]

  

Fig 2: VERNELL\_PRB\_CLSTR2: Random ES distribution      
 Gene set null distribution of ES for **VERNELL\_PRB\_CLSTR2**
